# Immunofluorescence Deconvolution Microscopy and Image Reconstruction of Human Defensins in Normal and Burned Skin

**Published:** 2005-04-25

**Authors:** Brian J. Poindexter

**Affiliations:** Multi-User Fluorescence Imaging and Microscopy Core Facility, Department of Pathology and Laboratory Medicine, University of Texas Medical School at Houston

## Abstract

**Objective:** The aim of this study was visualization and localization of the human antimicrobials human beta defensins 1, 2, and 3, neutrophil defensin alpha (human neutrophil peptide), and the cathelicidin LL-37 in normal and burned skin, and determination of the cell types in which these antimicrobials were localized. **Methods:** Tissue sections were probed with antimicrobial antibodies, tagged with fluorescently labeled secondary antibodies, and subjected to fluorescence deconvolution microscopy and image reconstruction. Images were generated by stacking multiple-section scans, which were then volume rendered by rotating stacks 360° about an axis, or modeled in 3 dimensions. **Results:** This technique yields a definitive image, providing a rapid basis for further quantification and manipulation from a full 3-dimensional aspect. In normal skin, human beta defensin-1 was localized to the perinuclear region of keratinocytes; human beta defensin-2 was primarily localized to the stratum germinativum; human beta defensin-3 was found in dendritic cells of the stratum spinosum; human neutrophil peptide was randomly distributed in the papillary dermis; and LL-37 was concentrated in the stratum corneum and along ducts. In burned skin, in which keratinocytes are lost or destroyed, human beta defensin-1 was present in dermal glandular structures including hair shafts; human beta defensin-2 and human beta defensin-3 were found in the remaining keratin layers and glands of the lower dermis; human neutrophil peptide was primarily localized to hair shafts, though visible in residual keratin layers; and LL-37 was evident in very high concentrations in the epithelium of sweat ducts. **Conclusion:** We conclude via this technique that cells in the lower dermal and subdermal regions of burned skin synthesize antimicrobials after burn injury, and maintain something of a barrier against infection. This methodology is discussed and explained in this article.

The skin has many natural defenses against infection. Tight junctions between keratinocytes prevent the invasion of microbes, and the fatty acid–rich and lipid-rich environment in the epidermis is toxic not only to bacteria but also to fungi and viruses.^1^ *Defensins* are natural antimicrobial peptides, produced by various cells in human skin,^2^,^3^ that also offer protection against invasion, especially when the skin barrier has been compromised by injury.^1^ Keratinocytes of the epidermis synthesize cathelicidins,^4^ eccrine glands produce sweat, which contains LL-37,^5^ mast cells produce LL-37,^4^ and neutrophils contain both human neutrophil peptides (HNPs) and LL-37.^6^ Therefore, the loss or destruction of skin removes many of our natural defense mechanisms.

We previously determined that natural antimicrobial peptides were present in burned skin,^7^,^8^ despite the loss of the epidermis and even the upper dermis, and localized these peptides to specific cell types and particular layers of the remaining skin.^9^ Fluorescence deconvolution microscopy yields definitive images that allow us to localize peptides and proteins to specific cell types and structures, and directs future studies to the upregulation of many of these microbials and the culture of multiple cell types for formulating wound cover matrices. Fluorescence deconvolution microscopy provides another research tool directed at the treatment of wounds and cellular regeneration.

## MATERIALS AND METHODS

All chemicals were purchased from Sigma Chemical Corp (St. Louis, Mo), except where stated, and were of the highest grade available.

### Tissue Preparation

Skin samples were obtained for frozen sectioning from patients admitted to the Regional Burn Center in Springfield, Ill, with partial- and full-thickness burns, ranging from 10% to 35% of total body surface area. Representative tissue specimens were harvested on the second or third day after injury during excision and grafting. Normal skin samples were taken from remnants of split-thickness autografts (0.30 mm). Samples were embedded in sucrose-based O.C.T. compound (Tissue-Tek, Torrence, Calif) and frozen on dry ice. Sections were cut at a thickness of 12 ± 3 μm with a Microm HM 505 E cryotome (Microm Laboratories, Walldorf, Germany) and placed on 18 mm glass cover slips (Fisher, Pittsburgh, Pa), which had been acid cleaned and coated with poly-l-lysine. Sections were fixed in 3.7% paraformaldehyde (Tousimis Research, Rockville, Md) for 5 minutes at room temperature, rinsed 5 times with phosphate buffered saline at room temperature, and cover slips were inverted and floated on 10% goat serum for 1 hour at 37°C to reduce nonspecific antibody binding. Antibodies for human defensins human beta defensin-1 (HBD-1), human beta defensin-2 (HBD-2), human neutrophil peptide-1 (HNP-1) (Alpha Diagnostic, San Antonio, Tex), human beta defensin-3 (HBD-3) (Novus Biologicals, Littleton, Colo), and LL-37 (Hycult Biotechnology b.v., Uden, The Netherlands) were diluted 1:100 in 10% goat serum and incubated with the sections for 45 minutes at 37°C. After rinsing the cover slips in 0.05% Tween-20 to remove unbound antibody, fluorescently tagged secondary antibodies (Molecular Probes, Eugene, Ore) were added and the sections were incubated for 30 minutes at 37°C. Finally, F-Actin and the nuclei were simultaneously fluorescently stained with phallicidin (Molecular Probes, Eugene, Ore) and 4′,6-diamidino-2-phenylindole (DAPI) (Molecular Probes) for 15 minutes at room temperature, and cover slips were mounted onto glass slides with Elvanol (DuPont, Willmington, Del) as the mounting media and attached with nail polish.

### Reconstructive Microscopy (Deconvolution)

Specimens were scanned with an Applied Precision DeltaVision (Issaquah, Wash) system fitted with an Olympus IX 70 inverted microscope employing a 100-W mercury arc lamp for illumination (Olympus America, Melville, NY) and excitation/emission filter sets (Chroma Technology Corp, Brattleboro, Vt) specific for each of the fluorescent antibodies. The filter set combination for DAPI (nucleus) was a 340 nm excitation filter with a band-pass of 20 nm and a 390 nm emission filter with a band-pass of 20 nm. Phallicidin (F-Actin) fluorescence was acquired with an excitation filter of 488 nm (band-pass 10 nm) and an emission filter of 520 nm (band-pass 25 nm). Defensin antibodies were visualized with an excitation filter of 585 nm (band-pass 10 nm) and an emission filter of 640 nm (band-pass 40 nm). Image scans for each probe were acquired in series at a step-size of 0.2 μm with a Sony Interline CCD camera. At least 30 sections were scanned per sample for each probe (ie, 90 total images for the 3 probes used). Specimen magnification was 400× unless otherwise noted.

Deconvolution and image analysis were performed by transferring the data sets to a Linux/RedHat workstation employing SoftWoRx software (Applied Precision) that uses an algorithm experimentally produced on the system from the convolution of a point spread function (PSF) to differentiate and reduce extraneous light or scattered light captured by the camera. A PSF describes the imaging and resolution characteristics of light collected by the optics of the microscope and was derived by scanning a 0.1 μm fluorescent bead (Molecular Probes, Eugene, Ore) 4 μm above and below the plane of focus. The resulting PSF was Fourier transformed into an optical transfer function that manipulated the data to produce images with a higher signal-to-noise resolution of the probe emission patterns. All data sets were subjected to 10 deconvolution iterations and then used for image analysis, image reconstructions, volume rendering, and modeling. Subtraction of background fluorescence and change of intensity gain were optimally set for each emission.

An image projection was produced by stacking each of the individual *z* sections of all 3 fluorescent probes into one image, resulting in a three-dimensional (3D) end product and an overlay of all colors. Volume rendering used the stack of *z* sections and rotated them about the *x* or *y* plane. Each of the volume rotation movies was produced with a 6° view angle. A 3D computer-generated virtual model of fluorescence emission patterns was produced to view the relative positions of each emission pattern and elucidate localization of the defensins to specific cells types. Briefly, positive and background intensities were measured and thresholds set to produce polygons based on the increased signal-to-noise ratio of the positive signal intensities. The software used these polygons to generate 3D objects and a virtual model that represented the emission patterns of each fluorescent probe. The 3D model was fully interactive and could be rotated in any plane for viewing at any angle or perspective.

The Institutional Review Boards of both Southern Illinois University and the University of Texas Health Science Center at Houston approved the study and appropriate consent was received.

## RESULTS

**Figure 1:**
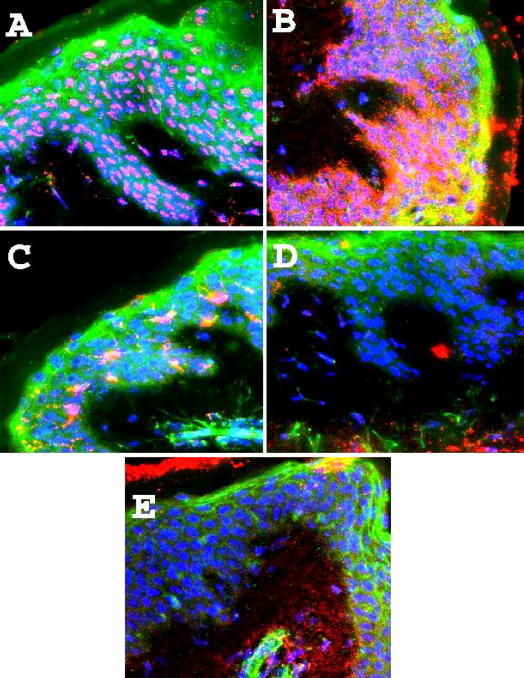
Antimicrobials in normal skin. A 5-panel collection of fluorescence images, obtained by deconvolution microscopy, at a magnification of 400× as described under the section “Materials and Methods.” All images are of normal skin samples and are probed for F-actin (green phallacidin), nuclei (blue DAPI), and the tagged secondary antibodies to the antimicrobials. Panel A is HBD-1 in keratinocytes; Panel B is HBD-2 in the stratum basale of the epidermis; Panel C is HBD-3 in dendritic cells; Panel D is HNP in the reticular layer of the dermis; and Panel E is LL-37 shown surrounding, and associated with, ducts.

Figure 1 shows stacked image acquisitions of the 5 antimicrobial peptides examined in sections of normal skin. Each image has been deconvoluted, the sections stacked, and the 3 colors overlaid (F-actin is green, nuclei are blue, and the defensins are red). Panel A shows HBD-1 localized to the keratinocytes, primarily in the peri-nuclear area; Panel B shows HBD-2 concentrated in the stratum basale, and distributed throughout the stratum spinosum and corneum; Panel C shows HBD-3 localized to dendritic cells; and Panel D is HNP, which was not found in the epidermis but was located in the upper levels of the dermis (determined by the presence of large cytoskeletal fibers). Finally, Panel E shows LL-37 associated with glands and ducts, absent from the spinosum but present in the outer corneum, indicating its deposition from secreted sweat.

**Figure 2:**
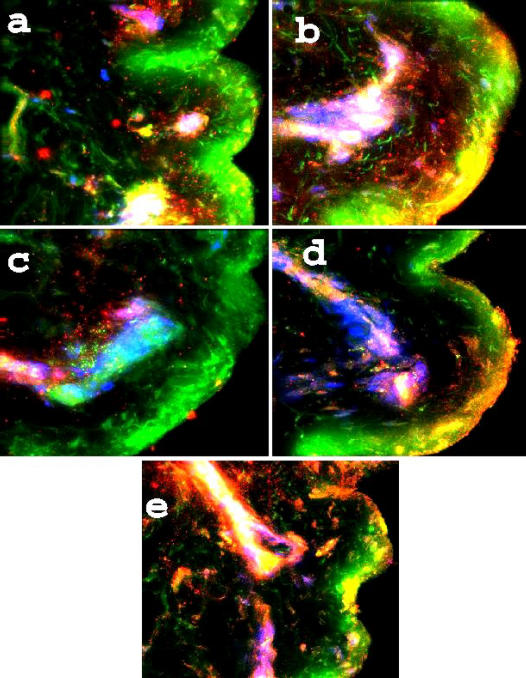
Antimicrobials in burned skin. A 5-panel collection of fluorescence images in burned skin samples. Magnification is 400×, and the probes are as used in Figure 1. Of note is the HBD-1 associated with dermal structures, not only in the upper levels but also with eccrine epithelia (a), HBD-2 associated with gland epithelia (b), HBD-3 in the lower papillary dermis (c), HNP along a hair follicle and on the upper, denatured keratin layer (d), and LL-37 very apparent via the bright yellow colocalization (green + red), along a sweat duct and deposited on the corneum at the point of exit.

Figure 2 is a compilation of selected images acquired from samples of burned skin taken from areas where there were portions of epidermis remaining. Each of the 5 panels demonstrates a loss of viable keratinocytes, a reduced definition of the layers, and a clumped, denatured appearance of the cytoskeletal elements. In panel a, because of loss of keratinocytes, there is no distinct nuclear pattern of HBD-1, though there are clumped masses in the upper papillary dermis and a cluster in the region of the reticular dermis. Panel b demonstrates localization of HBD-2 to eccrine glands, and in Panel c, HBD-3 is shown associated with vascular elements in some sections. Panel d is HNP and again demonstrates localization with a duct and some located diffusely throughout the upper dermis, while LL-37 (panel e) has outer corneal deposition and a high colocalization to duct epithelium. Ducts are distinguished from hair shafts by the lack of F-actin and connective tissue in the former.

**Figure 3:**
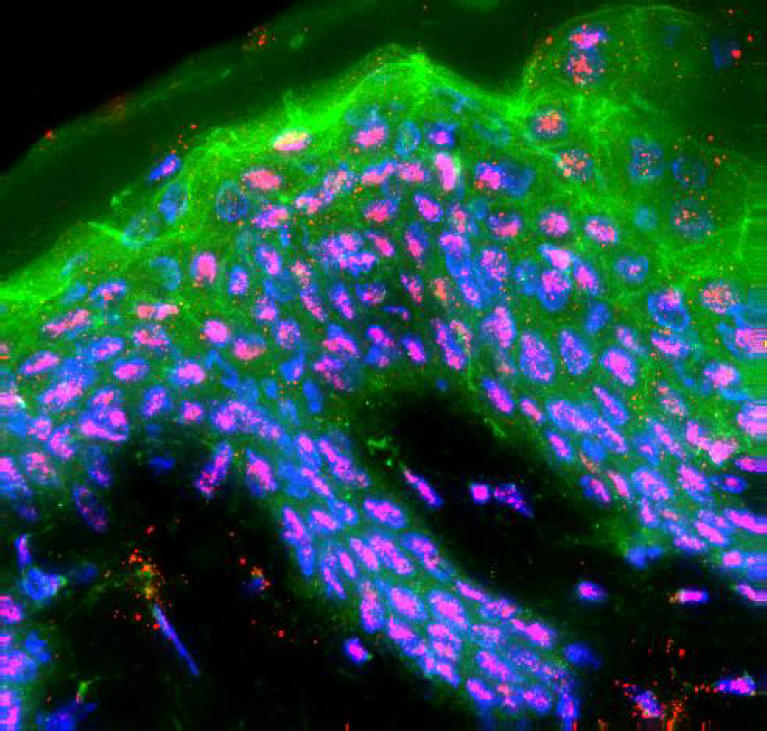
Volume rotation of HBD-1 in normal skin. This image of Panel A in Figure 1 highlights HBD-1 in normal skin and its localization to the nuclear region of keratinocytes. It also shows how abundant HBD-1 is in the epidermis. A movie of this image rotating is shown below.

Figure 3 is an example of volume rendering and volume rotation. This image of normal skin probed for HBD-1 rotates 360° about the *y* axis at a rotational angle of 6°. The rotation is of Panel A, in Figure 1, and demonstrates the specificity of HBD-1 to the nuclear region of keratinocytes. It also reveals the overall abundance and widespread distribution of HBD-1 in the epidermis, effecting protection against infection.

**Figure 4:**
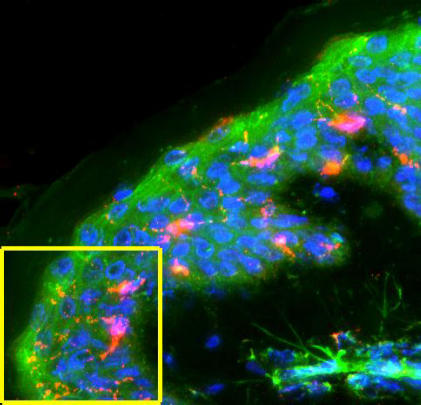
Volume rotation of HBD-3 in normal skin. This image of HBD-3 in normal skin (from Panel C in Figure 1) shows the area that is cut out and set to rotate in the following movie clip. HBD-3 shows a high specificity to dendritic cells of the epidermis. However, this 2-dimensional image is unable to fully display neither the 3-dimensionality of these dendritic cells nor the high specificity of HBD-3 to the specific cell type. Therefore, the following volume rotation clearly reveals this specificity and localization.

Figure 4 is another volume rotation of normal skin highlighting HBD-3. In normal skin, HBD-3 is predominantly found in the dendritic cells of the epidermis. The 2-dimensional image in Figure 1 (Panel C) does not fully display either the specificity of the peptide to the dendritic cells, or the 3-dimensional morphology of these dendritic cells. Therefore, Figure 4 shows the area of the image that is cut out, been volume rendered and set to rotate 360° about the *y* axis to better visualize the specificity of HBD-3.

**Figure 5:**
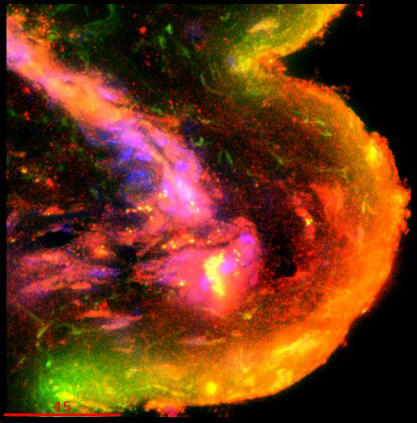
Volume rotation of HNP in burned skin. This image containing HNP in burned skin (Panel d in Fig 2) is set to rotate in the following video clip. The volume rotation demonstrates a loss of viable keratinocytes, but HNP is located throughout the upper dermis, and localized to a sweat duct.

Figure 5 is a volume rotation of burned skin probed for HNP, taken from Panel d in Figure 2. Rotation is 360° about the *x* axis. This image rotation clearly demonstrates a loss of viable keratinocytes but some HNP localization to the upper dermis. It also reveals some specificity of HNP to a sweat duct coursing through the upper part of the dermis. These volume rotations are not only useful for localizing positive emission patterns but also valuable for visualizing the true 3D nature of the specimens.

**Figure 6:**
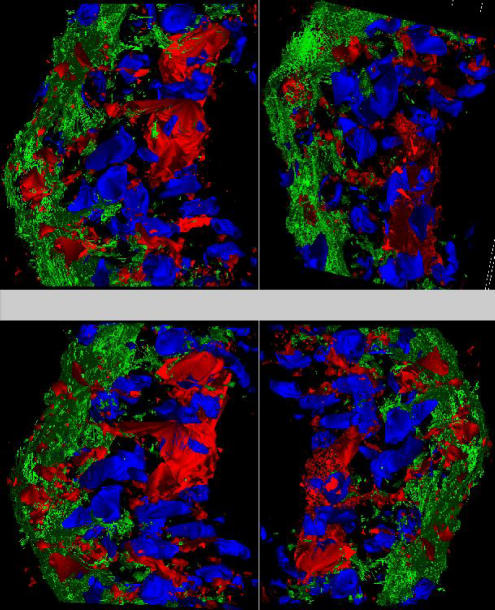
Virtual model of HBD-3 in normal skin. A 4-panel collection of images created from a computer-generated 3-dimensional virtual model of the cutout shown in Figure 4. Image zoom is 2×. Each image has been sequentially rotated in each of the 3 planes. Three-dimensional virtual model rendering highlights the specific 3-dimensional localization of HBD-3 in the dendritic cells.

Figure 6 shows images of a computer-generated model, with the colors consistent with previous images and figures. This method of volume rendering helps highlight the specific 3D localization pattern of HBD-3, and further demonstrates localization of HBD-3 to dendritic cells in the epidermis. Each of the images in the figure is produced by sequential rotation of the acquisition along the 3 axes.

## DISCUSSION

The presence of natural antimicrobials in skin plays an important role in the body's natural defenses, and the loss of these compounds reduces our ability to combat infection and sepsis.^1^,^10^ This is particularly true in cases of thermal injury, in which the resulting sepsis is often fatal.^10^ Thermal injury results in the removal or destruction of tight junctions and intercellular phospholipid-lipid milieu defenses. It would therefore be beneficial to determine which antimicrobials, if any, remain after burn injury has removed or destroyed the epidermal keratinocytes. In addition, determining which specific cell types continue to synthesize antimicrobials after burn injury would allow us to further cell culture research to identify a better matrix for application to wounds. Finally, it would also be beneficial to ascertain whether antimicrobial upregulation in remaining cells and structures after thermal damage is something that should be vigorously pursued.

Our earlier work^7^,^8^ demonstrated the loss of epidermal HBD-2 after thermal injury, even in small patches of the remaining epidermis. Also, defensin has been found to be absent from burn blister fluid.^11^ However, we reported that HBD-2 was located in deeper, dermal cells of burned skin, and this fact directed us to hypothesize that eccrine gland epithelia, or hair root–associated melanocytes, might be potential factories for antimicrobial production and that specific cell types could be cultured for production and harvesting of defensins to produce a topical agent, or wound cover matrix. Furthermore, upregulation of antimicrobial production by specific cells of the dermis would be a beneficial adjunct in the treatment of wounds and for combating infection.

In our recent studies, we imaged skin samples to localize natural antimicrobials before and after thermal injury to ascertain how much of this natural defense barrier against infection had been destroyed and to determine whether other cells and structures deeper to the skin should be considered for future cell culture and antimicrobial production experimentation. We employed fluorescence deconvolution microscopy to produce 3D, volume rendered images (computer-generated virtual models),^12^ which allowed us to determine in which specific cell type(s) each antimicrobial may be found. Imaging showed that much of this antimicrobial defense mechanism had been destroyed after thermal injury, with HBD-1 all but missing from the skin, except in clumped conglomerations near areas where Meissner's corpuscles might be found. HBD-2 gave a positive fluorescence in the reticular dermal layer, clustered around eccrine cells. HBD-3 was present in a clumped pattern, and seemed not to be associated with any particular cell type or dermal structure. HNP was located near larger vessels and along hair shafts, and LL-37 showed high intensity colocalizations with sweat ducts.

We therefore demonstrated that natural antimicrobials are synthesized in the deeper portions of burned skin and that cells other than keratinocytes that make these compounds are eccrine epithelia, duct epithelia, and cells found in the base of hair roots and hair bulbs. Thus, we may ask ourselves that when cells are cultured to produce a suitable wound covering matrix, should the coculture, and/or admixing, of other cell types with keratinocytes give us some basis toward a topical treatment in which multiple antimicrobials are produced, or are there drugs or chemicals that will allow us to target specific cell types to induce increased synthesis of these defensins in the remaining skin elements following burn injury?

This work gives us more insights into the role of antimicrobial peptides in burned skin and its protective mechanisms. This report also demonstrates the use of fluorescence microscopy not only in the research arena but also as a valuable and powerful tool in pathological studies. Questions remain regarding future studies into therapies that might be developed to combat burn sepsis and initiate a rapid healing response. Coupled with the ever-improving specificity of fluorescent probes and antibodies, and the continuing evolution of better microscopes, we should seriously consider more, everyday uses for fluorescence targeting.

## Figures and Tables

**Video 1:**
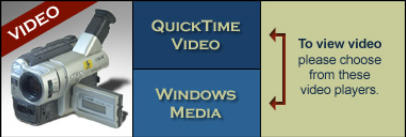
A volume rotation of Figure 3, rotating 360° about the *y* axis at a 6° viewing angle.

**Video 2:**
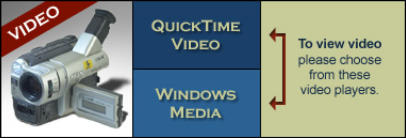
A volume rotation of only the area in the yellow box in Figure 4, rotating 360° about the *y* axis at a 6° viewing angle.

**Video 3:**
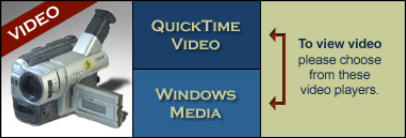
A volume rotation of Figure 5, rotating 360° about the *x* axis at a 6° viewing angle.
